# A new species of the genus *Dryinus* Latreille (Hymenoptera, Dryinidae) from the USA

**DOI:** 10.3897/zookeys.871.35974

**Published:** 2019-08-12

**Authors:** Stefano Speranza, Massimo Olmi, Adalgisa Guglielmino, Mario Contarini

**Affiliations:** 1 Department of Agriculture and Forest Sciences (DAFNE), University of Tuscia, Viterbo, Italy University of Tuscia Viterbo Italy; 2 Tropical Entomology Research Center, Viterbo, Italy Tropical Entomology Research Center Viterbo Italy

**Keywords:** Chrysidoidea, Dryininae, Georgia, Sapelo Island, taxonomy, key, Nearctic region, *Dryinus georgianus*, *Dryinus mexicanus*, *Dryinus splendidus*

## Abstract

A new species of *Dryinus* Latreille, 1804, is described from Georgia (USA). *D. georgianus***sp. nov**. is morphologically similar to *D. mexicanus* (Perkins, 1907) and *D. splendidus* Guglielmino and Olmi, 2013, but is distinguished by the lateral ocelli not touching the occipital carina (in the other two species, the lateral ocelli touch the occipital carina). The key to the females of the Nearctic species of *Dryinus* group 1 is modified to include the new taxon.

## Introduction

Dryinidae is a small family of Chrysidoidea (Hymenoptera) including 16 subfamilies, 50 genera, and approximately 1900 species worldwide (Olmi and Xu 2015; Tribull 2015; Olmi et al. 2019). The species of this family are parasitoids and often also predators of HemipteraAuchenorrhyncha (Guglielmino et al. 2013).

Dryinidae of the Nearctic region were studied mainly by Olmi (1984) and Guglielmino and Olmi (2013). In this region, the genus *Dryinus* Latreille, 1804, includes 20 species (Guglielmino and Olmi 2013; Olmi 1984, 1991, 1996, 2010, 2011; Ponomarenko 1981), among which only the following three species are recorded from Georgia, USA: *D. alatus* (Cresson, 1872) (Guglielmino and Olmi 2013; Olmi 1984), *D. testaceus* (Olmi, 1984) (unpublished record: Monroe Co., Forsyth, 1.VII.2000, 1 female in RAM), and *D. inconsultus* (Olmi, 1984) (unpublished record: Monroe Co., Forsyth, 18–23.V.1970, 1 female in CNC). All the above three species, including *D. alatus*, are known in Georgia only from one locality: Forsyth, in Monroe Co. In 2017 the authors examined a *Dryinus* specimen collected in Georgia and discovered a new species, described below.

## Materials and methods

The description follows the terminology used by Guglielmino et al. (2016a, 2016b, 2018) and Guglielmino and Olmi (2013). The measurements reported are relative, except for the total length (head to abdominal tip, without the antennae), which is expressed in millimetres. In the descriptions the following abbreviations are used:

**POL** distance between the inner edges of the lateral ocelli;

**OL** distance between the inner edges of a lateral ocellus and the median ocellus;

**OOL** distance from the outer edge of a lateral ocellus to the compound eye;

**OPL** distance from the posterior edge of a lateral ocellus to the occipital carina;

**TL** distance from the posterior edge of an eye to the occipital carina.

The term “metapectal-propodeal complex” is here used in the sense of Kawada et al. (2015). It corresponds to the term “metathorax + propodeum” sensu Olmi (1984), Olmi and Virla (2014), Olmi and Xu (2015) and Xu et al. (2013). The terms “metapectal-propodeal disc” and “propodeal declivity” sensu Kawada et al. (2015), used here, correspond to the terms “dorsal surface of propodeum” and “posterior surface of propodeum”, *sensu*Olmi (1984), Olmi and Virla (2014), Olmi and Xu (2015) and Xu et al. (2013).

The term “ADOs” (= Antennal Dorsal Organs) is here used in the sense of Riolo et al. (2016). It corresponds to the term “rhinaria” *sensu*Olmi (1984, 1994), Olmi and Virla (2014), Olmi and Xu (2015) and Xu et al. (2013). According to Riolo et al. (2016), ADOs are sensory structures, that might mediate the antennal responses to vibratory stimuli. As far as we know, they are present only in antennae of dryinid females attacking Fulgoromorpha (Olmi 1984, 1991, 1994).

The names of cells and veins of the forewing are here used in the sense of Azevedo et al. (2018). In all previous monographs on Dryinidae (Olmi 1984, 1994; Olmi and Virla 2014; Olmi and Xu 2015; Xu et al. 2013) different names were used. The correspondence between old and new names is the following (the first name is the old name): median cell = radial cell (R); submedian cell = first cubital cell (1Cu); stigmal vein = second radial cross & radial sector (2r-rs&Rs). In the text, cells and veins will be named by their respective abbreviations, including costal cell (C). The terminology of tegument sculpture follows Olmi and Virla (2014).

The types of all Nearctic species of *Dryinus* were examined. The holotype of the new species studied in this paper is deposited in the Royal Alberta Museum, Edmonton, Alberta, Canada (**RAM**). Other examined species from Georgia (USA) are deposited in the Canadian National Collection of Insects, Ottawa, Canada (**CNC**).

The description of the new species is based on the study of only a single specimen. The authors are aware that descriptions of new taxa should normally be based on more individuals. However, Dryinidae are so rare that it is uncommon to collect more than one specimen of each species. In addition, on the basis of the experience and knowledge of the authors, the new species is sufficiently delimited by unique characters to justify its description.

## Results

### Genus Dryinus

Taxon classificationAnimaliaHymenopteraPompilidae

Latreille, 1804

2bab3f93-dc2d-5bca-9e7d-d0c9f307f8e7

Dryinus Latreille, 1804: 176. Type species: Dryinus collaris Linnaeus, 1767, by subsequent monotypy (Latreille 1805).

#### Diagnosis.

Female: Fully winged; occipital carina complete, incomplete, or absent; palpal formula 6/3; mandible with 1–4 teeth; antenna usually with ADOs, rarely without, but always without tufts of long hairs; antennomere 1 longer than 2, variable, and antennomere 3 usually less than five times as long as antennomere 2, occasionally more than five times (in this case, notauli often complete, then mesoscutum completely sculptured by numerous and parallel longitudinal keels); pronotal tubercle reaching or not reaching tegula; forewing with three cells enclosed by pigmented veins (C, R, 1Cu); chela with rudimentary claw; protarsomere 5 less than twice as broad as enlarged claw; enlarged claw as long as, or shorter than protibia; tibial spurs 1/1/2, rarely 1/1/1. Male: Fully winged; occipital carina complete or incomplete; mandible with 1–3 teeth; palpal formula 6/3; epicnemium present and visible, because lateral regions of prothorax not continuous with mesopleura; mesosternum fused with mesopleura and not distinct; forewing with three cells enclosed by pigmented veins (C, R, 1Cu); paramere without dorsal process; tibial spurs 1/1/2.

### Dryinus georgianus sp. nov.

Taxon classificationAnimaliaHymenopteraPompilidae

530dfebe-77fa-5a3f-8dde-cf385f2149dc

http://zoobank.org/74BAFB91-5A11-4D9A-A41F-CAA905AA11C3

Figs 1 , 2

#### Diagnosis.

Female with body predominantly ferruginous, frontal line complete; OL shorter than POL; OL as long as TL; posterior ocelli not touching occipital carina; head and pronotum granulate; notauli complete; mesoscutum granulate and partly reticulate rugose, enlarged claw spatulate and with one tooth on lateral margin; Protarsomere 5 with approximately 35 lamellae.

#### Description.

**Figure 1.**
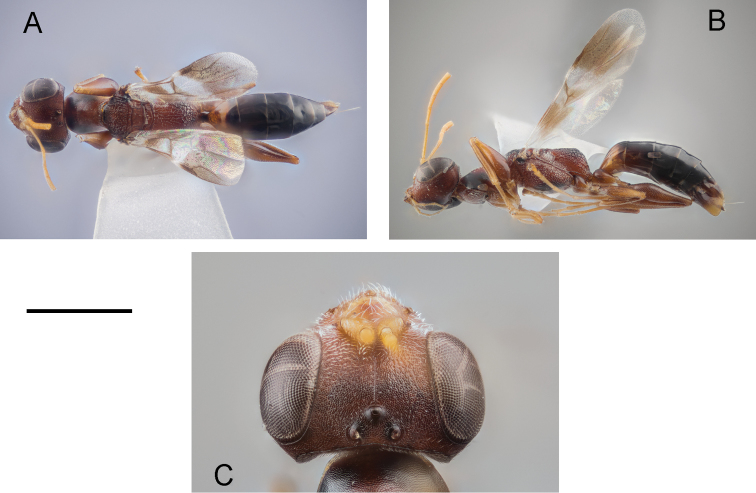
*Dryinus georgianus* sp. nov., female holotype: habitus in dorsal (**A**) and lateral (**B**) view; head in dorsal view (**C**). Scale bars: 1.28 mm (**A, B**); 0.46 mm (**C**).

**Figure 2.**
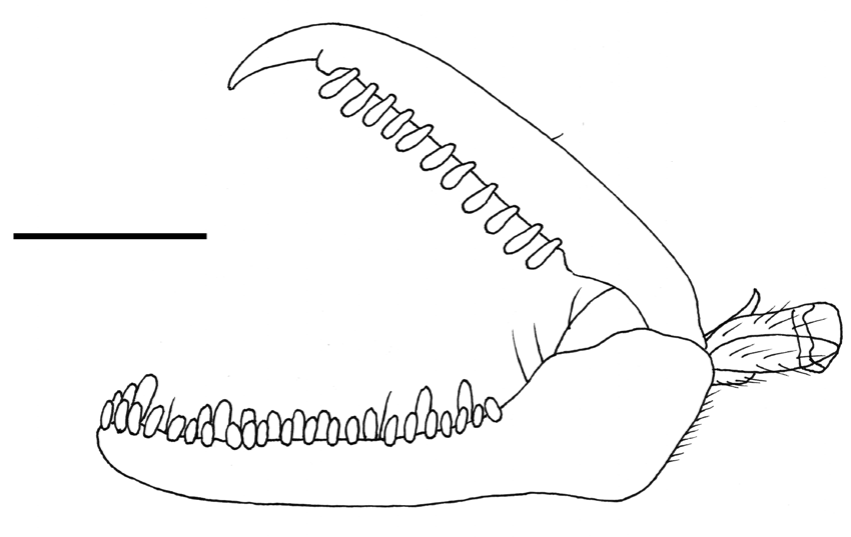
*Dryinus georgianus* sp. nov., female holotype: chela. Scale bar: 0.13 mm.

**Female** (Fig. 1A–C). Fully winged. Length 4.0 mm. Head and antenna ferruginous. Mesosoma ferruginous, except two lateral brown spots on pronotum. Anterior third and lateral regions of mesoscutum darkened. Part of lateral regions of metapectal-propodeal complex and propodeal declivity darkened. Petiole black. Metasoma brown, except first segment almost totally testaceous. Legs ferruginous. Antenna clavate. Antennomeres in following proportions: 9:4:18:9:7:7:6:6:5:8. ADOs present in antennomeres 5–10. Head (Fig. 1C) swollen, dull, granulate, except some irregular longitudinal keels on face. Frontal line complete. Occipital carina complete. Temple distinct. POL = 4; OL = 2; OOL = 7; OPL = 1; TL = 2. Greatest breadth of posterior ocelli about as long as OL. Pronotum dull, granulate, crossed by two transverse impressions, anterior one weak and posterior one strong. Disc of pronotum humped. Posterior collar of pronotum short, reticulate rugose. Pronotal tubercle not reaching tegula. Mesoscutum (Fig. 1A) dull, granulate, reticulate rugose on lateral regions and near posterior margin. Notauli complete, posteriorly separated, hardly visible near posterior margin of mesoscutum. Minimum distance between notauli about as long as POL. Mesoscutellum and metanotum dull, granulate. Metapectal-propodeal complex reticulate rugose, without longitudinal keels on propodeal declivity. Forewing (Fig. 1A, B) with three dark transverse bands. Distal part of 2r-rs&Rs vein much longer than proximal part (11:6). Protarsomere 3 produced into hook. Protarsomeres in following proportions: 17:2:3:11:17. Enlarged claw spatulate (Fig. 2), with one strong subapical tooth and one row of 11 lamellae. Protarsomere 5 (Fig. 2) with two rows of approximately 35 lamellae extending continuously to distal apex. Tibial spurs 1/1/2.

#### Male.

Unknown.

#### Material examined.

**Holotype**: female, USA: Georgia, McIntosh Co., Sapelo Island, 19.IX–19.XI.1987, FIT, shrub sand dunes, BRC Hym. Team (RAM).

#### Hosts.

Unknown.

#### Etymology.

The species is named *georgianus*, based on the collecting locality.

## Conclusions

The female of the new species has complete notauli (Fig. 1A) and enlarged claw provided with one strong subapical tooth (Fig. 2). Because of these characters, *D. georgianus* belongs to group 1 of *Dryinus*, according to the systematics proposed by Olmi and Virla (2014). In this species, the head is ferruginous (Fig. 1A, C); the mesoscutum is granulate and partly reticulate rugose, notauli complete (Fig. 1A) and the enlarged claw is spatulate (Fig. 2). Because of these characters, *D. georgianus* is similar to *D. mexicanus* (Perkins, 1907) and *D. splendidus* Guglielmino and Olmi, 2013, recorded respectively from Mexico and USA (Guglielmino and Olmi 2013). However, in *D. georgianus* the lateral ocelli do not touch the occipital carina (Fig. 2), whereas in the other two species they do. The key to the females of the Nearctic *Dryinus* group 1 presented by Guglielmino and Olmi (2013), should be modified by replacing couplet 8 as follows:

**Table:** 

8	Posterior ocelli not touching occipital carina (Fig. 1C)	***D. georgianus* sp. nov.**
–	Posterior ocelli touching occipital carina	**8**’
8’	Enlarged claw with lamellae very long (Guglielmino and Olmi 2013: fig. 10); protarsomere 5 with longer rows of lamellae (Guglielmino and Olmi 2013: fig. 10); protarsomere 1 about twice as long as 4; head with TL longer than POL	***D. mexicanus* (Perkins)**
–	Enlarged claw with shorter lamellae (Guglielmino and Olmi 2013: fig. 12); protarsomere 5 with shorter rows of lamellae (Guglielmino and Olmi 2013: fig. 10); protarsomere 1 less than 1.5 times as long as 4; head with TL shorter than POL	***D. splendidus* Guglielmino & Olmi**

Among the *Dryinus* species recorded from Georgia, *D. alatus* and *D. georgianus* belong to group 1, whereas *D. testaceus* and *D. inconsultus* belong respectively to groups 2 and 3.

*Dryinus* species are known to parasitize hosts belonging to the following families of Fulgoromorpha (Guglielmino et al. 2013): Acanaloniidae, Cixiidae, Dictyopharidae, Flatidae, Fulgoridae, Issidae, Lophopidae, Ricaniidae, Tropiduchidae. Larvae of *Dryinus* were described by Abril-Ramírez (1992) and Guglielmino et al. (2015).

## Supplementary Material

XML Treatment for Dryinus

XML Treatment for Dryinus georgianus
